# No aggression in a 4-year-old boy with an androgen-producing tumour: Case Report

**DOI:** 10.1186/1744-859X-4-17

**Published:** 2005-10-03

**Authors:** Wouter De la Marche, Karin Prinsen, Annemieke M Boot, Robert F Ferdinand

**Affiliations:** 1 Department of Child and Adolescent Psychiatry, Erasmus Medical Center Rotterdam/Sophia Children's Hospital, The Netherlands; 2 Department of Paediatrics, Erasmus Medical Center Rotterdam/Sophia Children's Hospital, The Netherlands

**Keywords:** androgens, testosterone, aggression, children

## Abstract

**Background:**

The androgen testosterone plays a critical role in many aspects of sexual differentiation. Also, it is thought to induce aggressive behaviours or to play a role in social dominance.

**Case presentation:**

In this case report a 4-year-old boy is described whose testosterone and dehydroepiandrosterone sulphate (DHEA-S) levels were raised to pubertal levels due to a testosterone producing testis tumour. This provided the unique opportunity to examine the effects of elevated levels of androgens on levels of aggression or on social dominance before the onset of puberty.

**Conclusion:**

The present case report does not support the hypothesis of a causal relationship between testosterone and aggression or between testosterone and social dominance in young children.

## Background

The androgen testosterone plays a critical role in many aspects of sexual differentiation. Also, it is thought to induce aggressive behaviours. Results of animal studies have indicated that, in most species, androgens, including testosterone, facilitate aggressive behaviour [1-5]. However, studies that investigated associations between androgens and aggression in humans have yielded inconclusive results and are very difficult to compare due to methodological differences. Moreover, most studies have compared hormone levels in individuals with high versus low aggression levels, or have yielded correlations between testosterone levels and the extent of aggression, which makes inferences about a causal relationship impossible. Furthermore, little is known about the relation between testosterone levels and the aggressive behaviour in pre-adolescent children. Because in normal development, testosterone levels are very low from the age of 6 months until pre-puberty, most studies have used samples that consisted of pubertal and post pubertal males. More recent studies hypothesised there might be an association between testosterone and social dominance instead [6-8]. Because ethically it is impossible to set up experiments to respond to the question of causal relationships, we should rely on natural experiments.

In the present report we describe the case of a 4-year-old boy whose testosterone and dehydroepiandrosterone sulphate (DHEA-S) levels were raised to pubertal levels due to pseudo pubertas praecox based on a testosterone producing testis tumour. This provided the unique opportunity to examine the effects of elevated levels of testosterone and DHEA-S on levels of aggression in a young child. The fact that the androgen levels in this boy were raised to about 30 times the normal level for his age makes this case a unique natural experiment.

## Case presentation

In June 2002, a boy aged 4 years and three months, was referred to the outpatient clinic of oncology of the Erasmus Medical Center of Rotterdam. One year before, penis growth had started to accelerate rapidly and 2 months prior to admission, his height had started to increase tremendously, pubic hair had started to grow, his voice had started breaking, and sweat production had increased. Furthermore, he often had erections during the day, got interested in older girls, and started reading magazines that contained pictures of naked women.

At clinical examination, his length was 120 cm (more than 2.5 SD above average) and he weighed 24.6 kg (weight/height ratio +1 SD). His sexual development had reached Tanner stages P3, G3 [9], with no underarm hair growth. Examination of his genitals showed a left testis of 2 ml and a right testis of 6 ml, firm and painless. Further clinical examination was negative, except for glue ears. X-rays of his hand showed that his skeletal age was 5 years ahead of his calendar age. His testosterone level (10.6 nmol/l) was raised to a pubertal level, as well as his DHEA-S level (1.30 μmol/l). Tumour markers carcino-embryonal antigen (CEA), alpha-fetoprotein, and beta human choriogonadotropin (β-HCG) were negative. Ultrasound of the testes showed a testicular tumour. Neither abdominal ultrasound nor abdominal CT or full bonescan (209MBq TC99M) showed signs of metastases.

Pseudo pubertas praecox based on a testicular tumour was diagnosed. After unilateral orchidectomy, pathological examination showed a Leydig Cell Tumour. One week after orchidectomy, hormone levels had normalized (testosterone < 0.10 nmol/l, DHEA-S < 0.20 μmol/l), he stopped having erections, and sweat production decreased, as did his sexual interest. His mother reported he had become more of a child again.

Because of anxiety, some behavioural problems, and delayed speech development, the boy was referred to our outpatient unit for child and adolescent psychiatry. At psychiatric examination, January 2003, we saw a boy who was anxious and withdrawn, had problems in social interaction, and a delayed speech development. Parents reported he had always been very quiet, however, since the beginning of 2002 he had started withdrawing himself more and more from social interactions, both at home and in school. To obtain standardized ratings of psychopathology, both parents, as well as the schoolteacher, were asked to fill out the Child Behaviour Checklist for ages 1 1/2–5 (CBCL 1 1/2–5) and the Caregiver-Teacher Report Form for ages 1 1/2–5 (C-TRF 1 1/2–5) [10]. The mother and the father scored the patient in the clinical range of the Withdrawn Behaviour, Anxious/Depressed Behaviour, and Somatic Complaint scales, but not on the Emotionally Reactive Behaviour, Sleep Problems, Attention Problems, and Aggressive Behaviour scales. The clinical cut-off corresponds with the 98^th ^percentile score in a general population sample [10]. Although the schoolteacher described the boy as quiet and withdrawn, and mentioned delayed speech development, C-TRF syndrome scores were not in the clinical range. No symptoms of externalizing behaviour (aggression, hyperactive behaviour or attention problems) were reported by the parents or the teacher, nor seen during psychiatric assessment. Further psychological testing showed that the boy's intellectual functioning was below average (Wechsler Preschool and Primary Scale of Intelligence – Revised [11]: Total IQ = 84; Verbal IQ = 82; Performance IQ = 92). DSM-IV [12] chronic adjustment disorder with anxiety was diagnosed.

## Discussion

In this case study, we report about a 4-year-old boy with an androgen producing testis tumour, in whom testosterone and DHEA-S levels rose to pubertal concentrations. It is a normal biological reaction that, when the testes begin to secrete androgens, sex drive and the motivation to seek sexual contact become stronger and are overtly expressed more and more [13,14]. It is remarkable however that, parallel to the sexual development and increased sexual drive, the boy we describe did not show increased levels of aggression. On the contrary, the boy was described as anxious and withdrawn.

The fact that the raised androgen levels are not accompanied by aggression is not consistent with the results of previous studies that found an association between androgen levels (testosterone and/or DHEA-S) and aggression [15-18]. For instance, in a study in which 8- to 12-year-old children (n = 15) with a Conduct Disorder were compared to normal controls (n = 25), van Goozen et al. [18] found significant correlations between DHEA-S levels and scores on the CBCL and TRF subscales Aggressive Behaviour (r = .46 and .48 respectively) and Delinquent Behaviour (r = .33 and .39 respectively). A possible explanation for the inconsistency between these studies and our 4-year-old boy can be that raised androgen levels are not the cause but a consequence of aggression.

Tremblay et al. concluded that testosterone at age 12 makes an independent contribution to explain social dominance at age 13. They consider as well that the high levels of testosterone could be as well the product as much as the cause of social dominance. They further suggest that the testosterone-dominance link should be present from infancy onwards, if it exists [8]. The boy we described in this case report had primarily high levels of testosterone, that didn't lead to social dominance. In contrast, he was anxious and withdrawn, which might have several causes. First, even without the occurrence of a hormone-producing tumour, the boy might have been at risk for these symptoms; family history was negative for anxiety but mother had suffered from a depressive episode before. Second, due to his extreme height, parents and other adults or children may have made age-inappropriate demands, which may have caused anxiety. Third, extreme changes in hormone levels may have directly resulted in withdrawal from social contacts. Sánchez-Martín et al. [19] examined 28 boys and 20 girls with a mean age of 4 years by videotaping them every morning during free play in the classroom during 4 months. They found that higher levels of testosterone were associated with decreased direct interaction with peers. The last explanation is in contradiction with the social dominance hypothesis.

**Figure 1:**
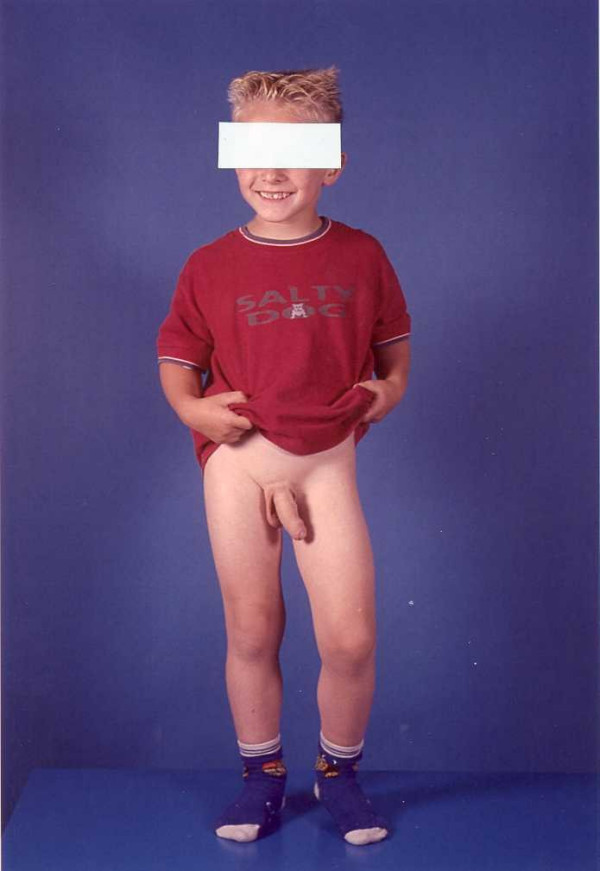
Age at the time of the picture: 4 years

Most of the studies that showed a relation between androgens and aggression were carried out with adults or adolescents. Previous studies with young children in prepuberty did not find much evidence for this relation [15,20]. For instance, Constantino et al. [20] studied 18 very aggressive 4- to 10-year-olds. They did not find an association between serum testosterone levels and the scores on the Aggressive Behaviour scale of the CBCL. To explain this lack of association, it can be argued that concentrations of androgens in young children are too low to find effects of testosterone on aggression anyway. In the boy in the present case report, however, serum testosterone levels were about 30 times the regular levels for his age and signs of aggression were still not found (figure 1).

## Conclusion

Although we know that one case cannot proof nor refute any hypothesis about causal relationships or associations between hormone levels and aggression or social dominance, natural experiments like this can help with formulating hypotheses for further research.

## Competing interests

The author(s) declare that they have no competing interests.

## Authors' contributions

Wouter De la Marche, MD: Mane author of the article.

Karin Prinsen, MSc, has made substantial contributions in writing the background and the psychiatric examination, as well as in revising the discussion.

Annemieke M. Boot, MD, PhD has carried out the somatic examination and supervised the technical examinations. She helped us formulating this part of the article. Robert F. Ferdinand, MD, PhD has been involved in revising this article critically for important intellectual content and has given final approval of this version of the article to be published.
